# Knowledge on added sugar content in food labels among adult out-patient clinic visitors at a tertiary care teaching hospital, Riyadh, KSA

**DOI:** 10.6026/97320630018455

**Published:** 2022-05-31

**Authors:** Kavita Sudersanadas, Maha Al Turki, Winnie Philip, Fawzia Alharbi, Dalal Almeqbel, Dalia Alanazi

**Affiliations:** 1 Dept. of Clinical Nutrition, College of Applied Medical Sciences, King Saud Bin Abdul-Aziz University for Health Sciences, National Guard Health Affairs, Riyadh, KSA -11481; 2 Research Unit, College of Applied Medical Sciences, King Saud Bin Abdul-Aziz University for Health Sciences, National Guard Health Affairs, Riyadh, KSA-11481; 3 King Abdullah International Medical Research Centre, National Guard Health Affairs, Riyadh, KSA-11481

**Keywords:** Knowledge, added sugar content, food labels, adult out-patient clinic visitors

## Abstract

Consumption of added sugars is reported as an etiological factor for high prevalence of diet-related diseases. Food labels of food products indicate the presence of added sugars. Knowing the different terms used to describe added sugars helps people to avoid food products rich in added sugars. Therefore, it is of interest to assess consumer knowledge about the added sugar terms on food labels. A study was conducted among 215 visitors of a tertiary care hospital outpatient clinic during July-September 2020. The data for this prospective cross-sectional study was collected by using online Google form. Adult visitors of both genders were selected using a non-probability convenient sampling technique. Demography and knowledge of the added sugars were collected. SPSS version 22 was used for data analysis. Mean (± SD), median, and Inter quartile Range (IQR), and Pearson Chi-square test were used. A "p" value of < 0.05 was considered statistically significant. The majority (96.7%) of the study participants was Saudi nationals with a median (IQR) age of 28 (23, 38). Most (68.37%) of the respondents were undergraduates. Physical inactivity (37.21%) and incidence of obesity (25.58%), and lifestyle diseases (15.40%) were reported. The chi-square test indicated a significant relationship between gender and knowledge of added sugars (χ2 = 69.85; p<0.05). Females (69.41%) have more knowledge about added sugars than males. These findings support the notion that there is a lack of knowledge about added sugar terms on the nutrition labels, which might contribute to the prevalence of obesity and other non-communicable chronic illnesses.

## Background:

Food labels are the prime important and direct means of communicating nutrition information to the customers. The internationally accepted definition of a food label is any tag, brand, mark, pictorial or other descriptive matter, written, printed, embossed or impressed on, stencilled, marked, or attached to, a container of food or foodstuff. Nutrition labelling has emerged as a preferred method for promoting healthy eating. It is viewed as a reliable source of information and a method to manipulate consumer behaviour at the point of purchase [1]. Food Labels help to assist customers in better identifying and using labels [2]. The increased prevalence of diet-related non-communicable diseases is one of the main drivers for nutrition labelling. FDA made food and nutrition labelling mandatory [3] so as to make the consumer aware about the advantages or drawbacks of the food ingredients on their health. Nutrition labelling required labelling all necessary components such as ingredients, carbohydrates, fat, protein, calories, minerals and vitamins [3,4]. Consuming a lot of sugars from drinks and food as well as lifestyle habits, may link to multiple health problems such as obesity, diabetes, dental cavities and heart disease [5]. Sugars are added to any recipe like a component added to recipe [6]. The added sugars make the diets energy dense [7]. The excess consumption of added sugars can be an etiological variable for the incidence of diet related non-communicable diseases such as cardiovascular diseases, stroke, diabetes, hyper cholesteremia, cancer, and obesity and dental caries [8]. The term "added sugar" generally refers to sugars (or ingredients that functionally replace sugars) that are added during preparation or processing to foods and beverages. Most popular added sugars are sugar, sweetener and syrup. The added sugars are also found in cane juice and cane syrup, corn sweeteners and high-fructose corn syrup (HFCS), fruit juice concentrate and nectars, honey, malt or maple syrup, molasses, soft drinks, canned juice, candy and desert [7]. The term added sugars do not include those sugars which found in food naturally. It was suggested that adults and children limit their free intake of sugar to less than 10% of the total daily intake of energy-equivalent to about 12 teaspoons [9]. As per dietary guidelines of Food and Drug Administration (FDA), food labels should include added sugars. In KSA, the responsibility of food labelling is vested on Saudi food and drug administration (SFDA). According to SFDA, all food products in Saudi Arabia must have food labelling to prevent or minimize the prevalence of diet related non-communicable diseases and to help people to make healthy dietary decisions [3]. Therefore, it is of interest the knowledge among adult out-patient clinic visitors of a tertiary care teaching hospital in Riyadh, KSA about the added sugar content on food labels.

## Materials and Methods:

The present study was conducted at the outpatient clinic of a tertiary care teaching hospital, King Abdulaziz Specialist Children's Hospital (KASCH) located in Riyadh city of KSA. Initially the method of data collection was face to face interview method. However, due to COVID 19 pandemic situation, the study was conducted by using GOOGLE forms through online.

## Study design/setting/sample selection:

This prospective cross sectional study was conducted among the patients who visited the hospital during the period from July to September 2020. The contact details of the patients were taken from electronic patient/medical records of the hospital. Non probability convenience sampling technique was used to select the respondents for the study.

## Subjects:

Two hundred and fifteen adults of age 18-50 years from both genders, who are willing to provide informed consent, were participated in the study.

## Data Collection Tools:

The data with respect to demography, health and nutritional status and knowledge of the respondents about added sugars were collected by using validated and reliable online google forms approved by the IRB of King Abdullah International Medical Research Centre. The forms consisted of both closed ended and open ended questions.

## Statistical analysis:

The data was analyzed by using SPSS version 22.The data from the Google forms were coded and edited before the data analysis. Categorical variables were analyzed by using frequencies and percentages. Distribution of continuous variables were expressed by mean (± SD) and skewed data were analyzed by using median and Interquartile Range (IQR).Pearson Chi square test was used to find the influence of demographic variables on the awareness of added sugar terms. A p value of < 0.05 was considered statistically significant.

## Results:

About 215 visitors of OP clinic of KASCH, who provided written signed informed consent partaken the study. The demographic characteristics of the respondents are provided in Table 1(see PDF). From Table 1(see PDF), it was observed that majority (96.7%) of the respondents are Saudi Nationalities with a median age of 28 years. Around 66% of the respondents were females. Many of the respondents have either under graduate (68.37%) or post graduate (10.2%) degrees. However, 60.9 % were unemployed and 32.6% of them had a family income ranging from 5001-10000SAR per month. The majority (37.21%) is physically inactive and led a sedentary lifestyle. Figure 1(see PDF) indicates the occurrence of lifestyle diseases among the respondents. It was perceived from the figure that most of the (54.42%) subjects were without any lifestyle diseases whereas 25.58% were obese, 7.96% had hyper cholesteremia and 7.44% were with Non-Insulin Dependent Diabetes Mellitus. The knowledge of the respondents based on nutrition science, about calorie and sugar intake and knowledge from nutrition labels were studied. The Knowledge of the respondents about added sugars based on the answers to knowledge-based questions about added sugars, by the respondents is given in Table 2(see PDF). From the table, it was observed that majority (91.16%) of the subjects' defined added sugar correctly. However, the awareness about recommendations of added sugars from various organizations such as AHA and WHO was less among the respondents. In this regards, the correct response was noted among 15.51 % and 13.49 % respectively for recommendations of WHO and AHA. Among the respondents, 17.39% aware about whose recommendation to reduce the added sugar intake. The data related to knowledge of respondents were categorized into three such as knowledge based on nutrition science, knowledge about sugar and calorie intake and knowledge based on nutrition labels and the results are presented in Table 2(see PDF). As depicted in Table 2(see PDF), female respondents have more knowledge about added sugars based on nutrition science(70.70%),knowledge about calorie and sugar intake(73%) and knowledge based on nutrition labels (64.83%) than their male counterparts. The study also showed that around 71% of the respondents are reading the nutrition labels however, 98.9 per cent of the respondents lack knowledge about variables related to calorie and sugar intake and 75.62 per cent of them deficient in knowledge based on nutrition science. The chi square test indicated that there was a significant relation between gender and knowledge of added sugars (χ2 = 69.85; p<0.05).The females have more knowledge about added sugars than males.

## Discussion:

Females formed the major proportion of the subjects of the study. Most of them were undergraduates. The family income of the majority of the subjects ranged from 5001-10000 SAR. It was found that most of the respondents were physically inactive (37.21 percent). High inactivity among the Saudi population was reported earlier also [10]. Earlier studies reported that, it is essential to be physically active to control the increased prevalence of NCD such as obesity, diabetes mellitus, cardio vascular diseases and cancer [11,12 and 13]. In addition, 40.98 % of respondents were self-reported to be suffering from lifestyle diseases. World Health Organization and American Heart Association recommend reducing the intake of free sugars [14,15]. However, the awareness about such recommendations was very low among the respondents of the study. Majority of the participants reported no awareness about the WHO (84.49%) and AHA (86.51%) guidelines. Females (70.4%) scored more than the males (29.6%).Such gender difference in the awareness about WHO guidelines about added sugars was also reported earlier [1]. The study also found that, 88.90% of the subjects had no knowledge about calorie and sugar intake. However, 71.47 % of the study participants can read and have certain information about added sugars given on the nutrition labels of the food products. The study, found that majority of the study participants (61.02 %) had no knowledge about added sugars as per the WHO and AHA guidelines, their calorie contribution as provided on the food labels. The study points out the need for nutrition education for different strata of the Saudi population. However, the current study was conducted on a convenient sample and hence, it is not a representative of the total population. Moreover, over representation of female (66.5%) and undergraduates (68.37%) are formed limitations for the study. Interestingly, even though the great majority of the participants were with higher levels of education, the proportion of participants with awareness about added sugars is very less. Hence, it can be assumed that the section of the people with lower levels of education had poor understanding about added sugars. Earlier studies reported that those from lower socioeconomic class have lower knowledge about nutrition [16]. Hence we recommend follow up study with a representative sample of the population. Moreover, a nation-wide educational program to help improving knowledge about added sugars and nutrition labels are to be initiated as an attempt to reduce the prevalence of chronic medical disease associated with added sugars.

## Conclusion:

Knowledge about the presence of added sugar to help consumers avoid unhealthy produces. This helps in reducing the prevalence of some of the common chronic medical disease conditions. Data documents awareness about added sugars was lower even among those who are undergraduates. The awareness about WHO and AHA guidelines was also found low. Hence, to translate and propagate the guidelines may be beneficial for the consumers. We suggest that public education programs targeting all individuals to improve the public awareness about the way to read food labels, how to identify and reduce the intake of added sugars. Such programs help to enable to reduce the prevalence of chronic medical disease associated with high intake of added sugars.
